# Contrasting symbolic and non-symbolic numerical representations in a joint classification task

**DOI:** 10.3758/s13423-023-02246-w

**Published:** 2023-01-17

**Authors:** Valter Prpic, Yasmine A. Basamh, Courtney M. Goodridge, Tiziano Agostini, Mauro Murgia

**Affiliations:** 1 Department of Philosophy and Communication Studies, University of Bologna, Via Azzo Gardino 23, Bologna, Italy; 2 Institute for Psychological Sciences, De Montfort University, Leicester, UK; 3 Institute for Transport Studies, University of Leeds, Leeds, UK; 4 Department of Life Sciences, University of Trieste, Trieste, Italy

**Keywords:** SNARC, Digit, Numerosity, Approximate number system, A theory of magnitude, Working memory

## Abstract

Both symbolic (digits) and non-symbolic (dots) numerals are spatially coded, with relatively small numbers being responded faster with a left key and large numbers being responded faster with a right key (spatial–numerical association of response codes [SNARC]). The idea of format independent SNARC seems to support the existence of a common system for symbolic and non-symbolic numerical representations, although evidence in the field is still mixed. The aim of the present study is to investigate whether symbolic and non-symbolic numerals interact in the SNARC effect when both information is simultaneously displayed. To do so, participants were presented with dice-like patterns, with digits being used instead of dots. In two separate magnitude classification tasks, participants had to respond either to the number of digits presented on the screen or to their numerical size. In the non-symbolic task, they had to judge whether the digits on the screen were more or less than three, irrespective of the numerical value of the digits. In the symbolic task, participants had to judge whether the digits on the screen were numerically smaller or larger than three, irrespective of the number of digits being present. The results show a consistent SNARC effect in the symbolic task and no effect in the non-symbolic one. Furthermore, congruency between symbolic and non-symbolic numerals did not modulate the response patterns, thus supporting the idea of independent representations and questioning some propositions of current theoretical accounts.

**Supplementary Information:**

The online version contains supplementary material available at 10.3758/s13423-023-02246-w.

## Introduction

Previous studies suggest that people represent numbers spatially resembling a mental number line (Restle, 1970; for a review, see Toomarian & Hubbard, 2018). A consistently replicated phenomenon that supports this view is the spatial–numerical association of response codes (SNARC) effect (Dehaene et al., 1993). This consists of faster left key responses for small numbers and faster right key responses for large numbers. The direction of this representation seems to be culturally determined, with reading and writing direction as well as finger counting being considered as the basis for this long-term association (Fischer & Shaki, 2017; Pitt & Casasanto, 2020; Shaki et al., 2009). However, it has been shown that contextual manipulations can reverse the direction of the spatial–numerical association (Bächtold et al., 1998; Mingolo et al., 2021).

Although symbolic numerals have been the most investigated stimuli, SNARC-like effects have been revealed in a multitude of non-numerical dimensions. While examples of symbolic non-numerical stimuli are relatively rare and can be found in music notation (Ariga & Saito, 2019; Fumarola et al., 2020; Prpic et al., 2016) and letters of the alphabet (Gevers et al., 2003), non-symbolic stimuli have been widely studied across different modalities. Most common examples are in the visual modality with the size of pictorial figures (Prpic et al., 2020; Ren et al., 2011), luminance (Fumarola et al., 2014; Ren et al., 2011), angle magnitude (Fumarola et al., 2016) as well as emotional magnitude in facial displays (Holmes et al., 2019; Holmes & Lourenco, 2011; but see also Baldassi et al., 2021; Fantoni et al., 2019). There are also numerous examples in the auditory modality with pitch (Lega et al., 2020; Lidji et al., 2007; Pitteri et al., 2015; Prpic & Domijan, 2018; Rusconi et al., 2006), loudness (Bruzzi et al., 2017; Hartmann & Mast, 2017), and temporal aspects of the auditory stimuli (De Tommaso & Prpic, 2020; Ishihara et al., 2008; Mariconda et al., 2022) being commonly investigated. Recently, somatosensory information has been studied revealing similar effects for weight (Dalmaso & Vicovaro, 2019; Vicovaro & Dalmaso, 2020) and vibrotactile stimuli (Bollini et al., 2020).

The ATOM (a theory of magnitude) model (Bueti & Walsh, 2009; Walsh, 2003) has been commonly used as a framework to encompass a whole range of SNARC-like effects since the theory posits that space and quantity are processed by a generalized magnitude system. Walsh (2003) also suggested that SNARC should prove to be a SQUARC (spatial–quantity association of response codes) effect whereby magnitudes across different domains should be spatially coded similarly to numbers. The presence of SNARC-like effects for a large variety of magnitude related stimuli seems to support Walsh’s (Walsh, 2003) prediction, although it is still a matter of debate whether these effects are driven by stimulus magnitude or ordinality (see Casasanto & Pitt, 2019; Prpic et al., 2021). Indeed, while the ATOM model focuses on the magnitude properties of the stimuli, the working memory (WM) model (van Dijck & Fias, 2011) claims that all stimuli can be spatially organized in WM and can elicit SNARC-like effects. Thus, both models fundamentally suggest that numbers have no special relationship with space and that numerical and non-numerical stimuli should elicit comparable SNARC-like effects. Conversely, a recent review and meta-analysis (Macnamara et al., 2018) established that the effect size for non-numerical domains (e.g., temporal, musical, size) is substantially smaller than the reported effect size for symbolic numerals (Wood et al., 2008). This evidence adds to other studies suggesting that numbers, specifically in their symbolic format, are fundamentally different from other ordinal or magnitude related stimuli (Dodd et al., 2008; Kadosh et al., 2007; Kadosh & Walsh, 2009).

Non-symbolic numerals (or numerosity) are less studied than their symbolic counterpart, however they recently gained renewed interest. To our knowledge, Nuerk et al. (2005) published the first study that investigated the SNARC effect for dot patterns. This study used configurations of dots resembling dice patterns and showed that small (vs. large) numerals are responded faster with a left (vs. right) key, independently from the format of numerical stimuli. More recently, this finding has been replicated by using randomly distributed dot clouds with larger numerosities (Nemeh et al., 2018; Zhou et al., 2016). Another recent study (Cutini et al., 2019) specifically focused on stimulus arrangements and revealed that both structured and unstructured patterns elicit a consistent SNARC effect in a small numerosity range (i.e., 1–9). These studies suggest that the SNARC effect for non-symbolic numerals is independent from both the range and the spatial arrangement of the stimuli.

Evidence of format independent SNARC effects supports the existence of a common system for symbolic and non-symbolic number processing. Traditionally, it has been considered that both numerical formats share the same neural representation (approximate number system, or ANS) and that non-symbolic numerals provide a foundation for their symbolic counterparts (Dehaene, 1993; Nieder, 2016; Nieder & Dehaene, 2009; Piazza, 2011; Piazza et al., 2007). However, several recent studies that have been questioning the existence of ANS (Núñez, 2017; Van Hoogmoed et al., 2021; Van Hoogmoed & Kroesbergen, 2018). Growing empirical evidence suggests a fundamental distinction between symbolic and non-symbolic numerals that challenges the idea of a common system for representing and processing these two numerical formats (Algom, 2021; Bar et al., 2019). In sum, symbolic numerals are deemed to be represented in a linear fashion, while non-symbolic numerals in a logarithmic fashion. Thus, psychophysical laws only apply to non-symbolic numerals, which are processed in the same way as all other perceptual continua (such as loudness or brightness), while symbolic numerals are processed in a unique and exact way. The assumption, proposed by ANS, that non-symbolic numerals are somehow unique and different from other perceptual continua has been challenged, and consequently the idea that a dedicated number system is needed to process and represent numerosity (Núñez, 2017).

Evidence from studies that compared the SNARC effect for symbolic and non-symbolic numerals are scarce; thus, no contribution in the ANS debate was provided from this line of research. A study that showed a SNARC effect for both symbolic and non-symbolic numerals in either adults or children managed to demonstrate that the two effects are not correlated, thus suggesting that symbolic and non-symbolic numerals are independently associated with space (He et al., 2021). Although behavioural evidence is still mixed, a growing number of research seem to be in favour of a dissociation for symbolic and non-symbolic numerical representations, at least for studies using SNARC paradigms (for a review, see Buijsman & Tirado, 2019). However, a limitation of previous studies that compared symbolic and non-symbolic SNARC effects is that these were tested separately, thus not allowing to directly assess whether these two representations interact.

Although symbolic and non-symbolic numerals have been previously investigated in combined settings (e.g., Pansky & Algom, 2002), the present work is the first attempt to directly contrast the spatial–numerical association for symbolic and non-symbolic numerals by presenting both stimuli simultaneously. To do so, we created dice-like patterns, but instead of dots, we displayed digits. In two separate tasks, participants were required to either respond to the symbolic value of the digits whilst ignoring their numerosity, or to respond to the number of digits present whilst ignoring their symbolic value. According to previous literature, both symbolic and non-symbolic numerals should elicit a SNARC effect. Therefore, when judging non-symbolic numerals, small (vs. large) numerosity should elicit faster left (vs. right) responses. However, since symbolic numerals are also simultaneously present with numerosity, these should also elicit a SNARC effect despite being task irrelevant (Fias et al., 2001). The same should work in the other direction, although evidence of non-symbolic numerals eliciting SNARC effects when numerical magnitude is task irrelevant are scarce (for an example, see Nuerk et al., 2005, and Mitchell et al., 2012).

If both symbolic and non-symbolic numerals are represented by a shared system, we would expect the SNARC effects to positively interact in the congruent condition (small digits/small numerosity; large digits/large numerosity), leading to a stronger spatial–numerical association. Similarly, in the incongruent condition (small digits/large numerosity; large digits/small numerosity), we should expect the SNARC effects to negatively interact as the effects for symbolic and non-symbolic numerals would have opposite directions. In this condition we would expect an absent or weak SNARC effect. Conversely, if these two representations are independent, as suggested by recent evidence (Buijsman & Tirado, 2019; Marinova et al., 2021; Sasanguie et al., 2015), congruency between symbolic and non-symbolic numerals should not impact the SNARC effect.

## Method

### Participants

An a priori power analysis was conducted using the software MorePower 6.0.4. Based on a recent study that investigated the SNARC effect for non-symbolic numerals (Cutini et al., 2019), we set the following parameters: power = .80, α = .05, partial eta squared = .21 for repeated-measures analyses of variance (ANOVAs); power = .80, α = .05, Cohen’s *d* = .43 for one-sample *t* tests. The largest sample size suggested by the two tests was 44. We decided to be more conservative and considered a sample of approximately 50 participants to be adequate.

Fifty-two students (48 females) from De Montfort University took part in the study and were rewarded with coursework credits. The mean age was 21.0 years (*SD* = 4.7). Forty-one participants were right-handed, whilst seven were left-handed. All participants reported to have normal or corrected-to-normal vision and were naïve about the purpose of the study. Written informed consent was obtained before participation. The study was approved by the Faculty of Health and Life Sciences Research Ethics Committee (Ref: 3488) and was conducted in accordance with the ethical standards established by the Declaration of Helsinki.

### Apparatus and stimuli

The online experiment was designed using PsychoPy (Version 2020.2.5; Peirce et al., 2019), and then conducted on Pavlovia through the participants’ personal computers. Responses were collected using the ‘A’ and ‘L’ keys on the participants’ computer ‘qwerty’ keyboards.

**Fig. 1:**
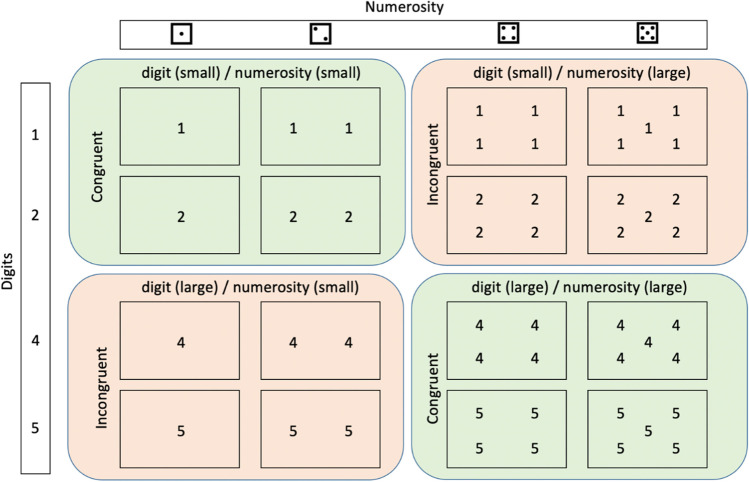
A depiction of the stimuli and the experimental manipulations. Symbolic (digits) and non-symbolic (numerosity) numerals were combined to create congruent (small digit/small numerosity; large digit/large numerosity) and incongruent (small digit/large numerosity; large digit/small numerosity) conditions

Stimuli consisted of four digits (1, 2, 4, and 5) presented in white against a grey background with the letter height set at 0.08 height units. Each trial presented only one number out of the four, and in each trial, the numbers were displayed as a dice-like formation (see Fig. 1). When only one number was shown, it was positioned in the centre of the screen (0, 0), two numbers were positioned with the coordinates (−.08, 0) and (.08, 0), four numbers were positioned at (−.08, .08), (.08, .08), (−.08, −.08), and (.08, −.08), whilst five numbers were positioned at (0, 0), (−.08, .08), (.08, .08), (−.08, −.08), and (.08, −.08). The range and the spatial arrangement of the stimuli were chosen to mimic the mapping of non-symbolic quantities in typical dice patterns. Between each trial, there was a fixation cross set at the centre of the screen with a height of 0.1.

### Procedure

The experiment took place online and participants were required to complete the experiment in a quiet room without distractions. Participants were instructed to place their right index finger on the rightmost key ‘A’ and their left index finger on the leftmost key ‘L’. Each trial started with a blank screen (500-ms duration), followed by a fixation cross (500-ms duration) and another blank screen (500-ms duration); 1,500 ms after the start of the trial, the target numbers were presented for 3,000 ms, and participants were allowed to respond till stimuli elapsed. All participants completed two tasks that had two separate blocks of trials each. In the symbolic task, participants were required to only judge symbolic numerals (digits) and ignore non-symbolic numerals (numerosity). They had to determine if the digit was larger or smaller than the reference standard (3); when the digit was larger, participants had to press the ‘L’ key; when the digit was smaller, participants had to press the ‘A’ key. After completing the first block in the symbolic task, the keys were switched for the second block; if the digit was smaller, the ‘L’ key was pressed, and if the digit was larger, the ‘A’ key was pressed. The instructions were the same for the non-symbolic task, where the task required the participant to determine whether there were more or less than three digits on the screen (non-symbolic numeral/numerosity) while ignoring the digits’ magnitude (symbolic numerals). Similar to the symbolic task, the response keys for the second block of the non-symbolic task were switched.

Participants were randomly split into four groups where the order of the two tasks and their consequent blocks was counterbalanced across all participants. Each block started with 16 practice trials before the participant completed 80 trials for the main trials. Trials in each block were randomized and all four numbers were equally presented in each of the four dice-like positions. Additionally, there were an equal number of ‘smaller’/‘larger’ responses in each block. This resulted in each participant completing 320 main trials. Participants were allowed a break between each block until they were ready to continue to the next block. Both speed and accuracy of responses were stressed in the instructions.

### Data preparation

Reaction times less than 150 ms were removed (Brenner & Smeets, 1997). Data from two participants were also removed for having a high number of errors (over 20%). The remaining sample made few errors (0.93%–13.47%), with average error percentage being 5.70%. Because of this, accuracy was not analysed. Thirty-four trials where participants failed to make a response were removed from the analysis alongside all incorrect responses. For outliers, we specified a threshold of 3 standard deviations and calculated the individual means and standard deviations within each condition. This approach detected 304 outlier trials, which were removed from the sample before analysis. The average number of outliers per participant was 5.73 (minimum was 1 and the maximum was 14). Data and analysis scripts are available on the Open Science Framework (https://osf.io/e7rj3/).

## Results

### Symbolic task

**Fig. 2:**
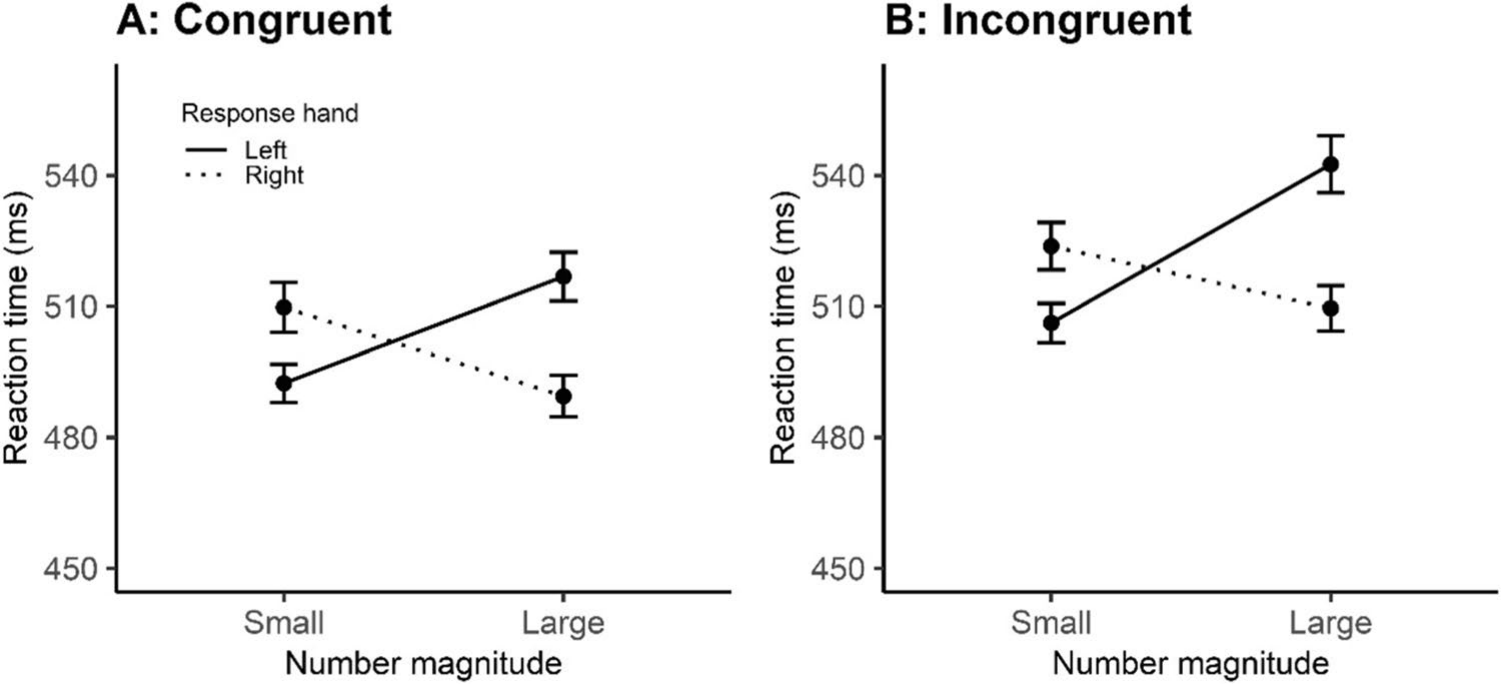
Mean reaction times, with error bars representing standard error of the mean (*SEM*), for congruent (**A**) and incongruent (**B**) conditions in the symbolic task

Individual mean reaction times (see descriptive data in Appendix, Table 1) were entered into a response hand (left vs. right) × number magnitude (small vs. large) × congruency (congruent vs. incongruent) repeated-measures ANOVA. A main effect of congruency *F*(1, 51) = 25.06, *p* < .001, *η*_*p*_^2^= .330, was found, suggesting that reaction times were faster when the numerical information was congruent (*M* = 502.06, *SE* = 2.56) versus incongruent (*M* = 520.39, *SE* = 2.73) (small/large digits were presented in small/large numerosity). A main effect of magnitude *F*(1, 51) = 4.96, *p* = .03, *η*_*p*_^2^= .089, was also found, suggesting that participants were faster in responding to smaller numerical magnitude (*M* = 507.98, *SE* = 2.51) in comparison to larger numerical magnitudes (*M* = 514.38, *SE* = 507.98). Most importantly, a significant Hand × Magnitude interaction was found *F*(1, 51) = 7.53, *p* = 0.008, *η*_*p*_^2^ = .129, which is clear evidence of a SNARC effect (Fig. 2). No other interactions were significant and there was no evidence of a three-way interaction between hand, magnitude, and congruency, suggesting that the SNARC effect was not modulated by congruent/incongruent non-symbolic numerals.

### Non-symbolic task

**Fig. 3:**
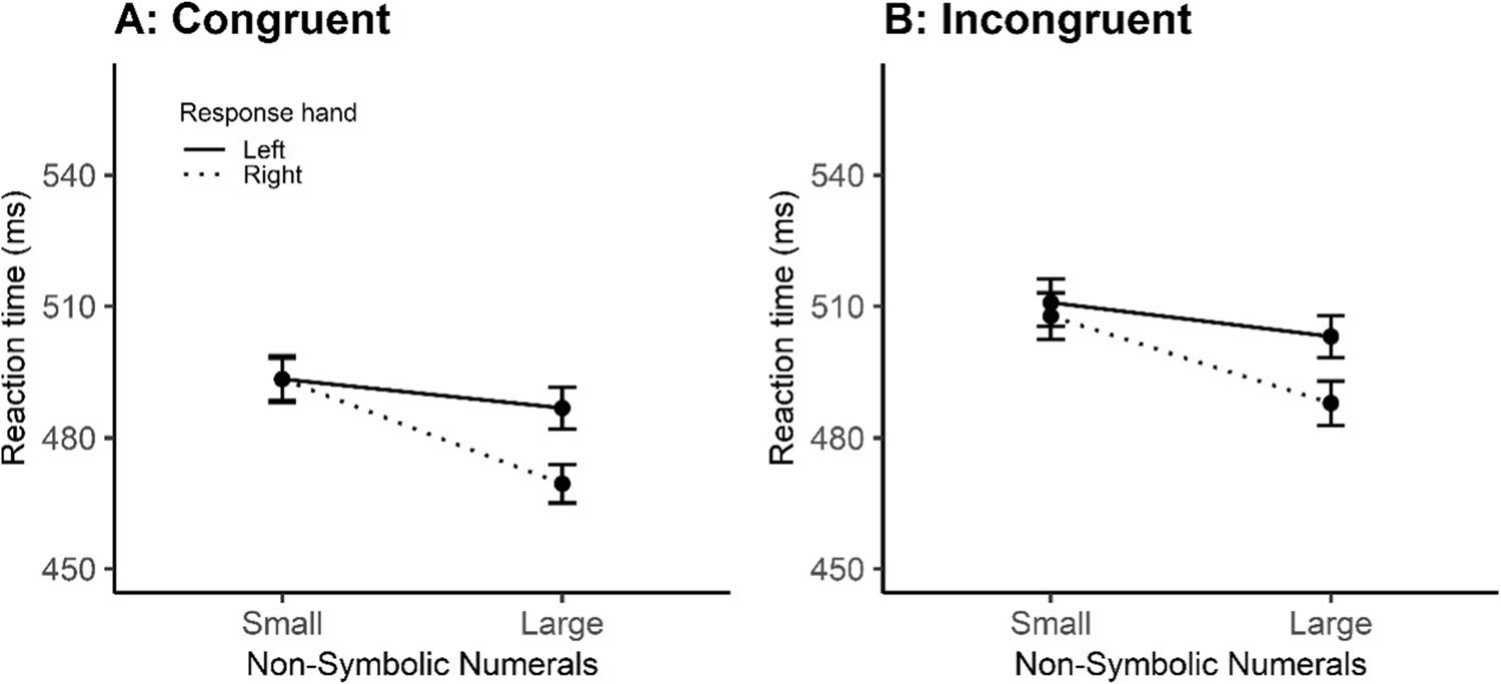
Mean reaction times, with error bars representing *SEM* for congruent (**A**) and incongruent (**B**) conditions in the non-symbolic task

Individual mean reaction times for the numerosity task (see descriptive data in Appendix, Table 2) were entered into a response hand (left vs. right) × non-symbolic numerical magnitude (small vs. large) × congruency (congruent vs. incongruent) repeated-measures ANOVA. A significant main effect of congruency was found, *F*(1, 51) = 28.44, *p* < .001, *η*_*p*_^2^ = .358. Once again, this suggests that participants were faster to react when the numerosity stimuli were congruent (*M* = 485.68, *SE* = 2.42) versus incongruent (*M* = 502.34, *SE* = 2.58). We also found a significant main effect of response hand, *F*(1, 51) = 5.13, *p* = 002, *η*_*p*_^2^ = .091. This suggests that participants were significantly faster at responding when using their right (*M* = 489.35, *SE* = 2.53) versus left (*M* = 498.35, *SE* = 2.47) hand. Finally, we found a significant main effect of magnitude, whereby responses to large magnitudes (*M* = 486.57, *SE* = 2.38) were faster than small magnitudes (*M* = 501.18, *SE* = 2.61), *F*(1, 51) = 13.32, *p* < .001, *η*_*p*_^2^ = .207. However, no interactions were significant in the ANOVA, and therefore there was no evidence of a SNARC effect (Hand × Magnitude interaction), *F*(1, 51) = 0.38, *p* = .542, *η*_*p*_^2^ = .007) (Fig. 3).

## Discussion

The aim of this study was to directly contrast the SNARC effect for symbolic and non-symbolic numerals within the same experiment. To do so, we created a novel set of stimuli consisting of dice-like patterns with digits being displayed instead of dots. Therefore, both symbolic (numerical value of digits) and non-symbolic (number of digits on the screen) numerals were simultaneously present within the same stimuli. This resulted in congruent (small numerosity/small digits or large numerosity/large digits) and incongruent (small numerosity/large digits or large numerosity/small digits) conditions. Participants were required to perform a symbolic and a non-symbolic magnitude classification task in separate sessions, while all other variables were kept constant. Based on both ANS and ATOM, we should expect that congruency between symbolic and non-symbolic numerals would interact with the SNARC effect; conversely, our results are in line with recent evidence suggesting independent representations for symbolic and non-symbolic numerals (Buijsman & Tirado, 2019; Marinova et al., 2021; Sasanguie et al., 2015).

When participants were required to process symbolic numerals a robust SNARC effect was found, with small digits being responded to faster with left key presses, and large digits with right key presses. Contrary to what should be expected from a shared numerical representation, symbolic and non-symbolic numerals did not interact. More specifically, non-symbolic numerals neither facilitated nor inhibited the SNARC effect in the congruent and incongruent conditions, respectively, thus supporting the idea of independent representations. Although we cannot exclude that non-symbolic numerals did not interact with the SNARC effect simply because they were task irrelevant (see Cleland et al., 2020 ; Pellegrino et al., 2021), our data clearly show overall slower response times in the incongruent conditions. This indicates that, despite being task irrelevant, numerosity was still processed and did impact participants’ responses, but did not interact with the SNARC effect.

When participants were required to process non-symbolic numerals, a SNARC effect was not detected. This is in contrast with previous studies that revealed a SNARC effect for dots arranged either as dice patterns (Cutini et al., 2019; Nuerk et al., 2005) or distributed randomly in the visual field (Cutini et al., 2019; Nemeh et al., 2018; Zhou et al., 2016). Furthermore, Mitchell et al. (2012) showed that the SNARC effect for numerosity is stronger in the subitizing range, thus suggesting that the absence of the effect cannot be due to the range of the stimuli employed in our study. The absence of a SNARC effect for numerosity might be ascribed to our ‘atypical’ non-symbolic stimuli which contained symbolic numerals instead of dots. However, if the symbolic nature of the stimuli were responsible for this result, we should expect a SNARC effect to be driven by digit magnitude which is known to elicit SNARC effects even when task irrelevant (e.g., Fias et al., 2001). Conversely, our data show that digits did not modulate the response pattern for non-symbolic numerals. However, similar to the symbolic task, slower responses were detected in the incongruent condition suggesting that irrelevant symbolic numerals were still processed during the task.

Our results for numerosity judgment add to recent evidence suggesting that, differently from digits, non-symbolic numerals do not offer a direct route to spatial–numerical associations (Cleland et al., 2020; Pellegrino et al., 2021). Furthermore, this evidence questions the ATOM model (Walsh, 2003) which posits that magnitudes across different domains and formats should be spatially coded similarly to digits. Conversely, in our study, SNARC seems to be closely related to symbolic numerals, thus failing to prove to be a SQUARC effect as predicted by Walsh (2003). Finally, our findings also challenge the WM model (van Dijck & Fias, 2011). Indeed, this account posits that every type of stimuli can be spatially organized in WM during task execution and, consequently, can elicit a SNARC-like effect (first items of the sequence are associated with left responses and later items with the right, independently from their identity). Therefore, similar SNARC effects should be elicited by both symbolic and non-symbolic numerals, while a clear and consistent difference emerged in our study.

Taken together, the facts that (1) non-symbolic numerals did not modulate the SNARC effect for digits and (2) symbolic numerals did not interact with the response pattern for numerosity, are in contrast with the idea of a common system for number processing (ANS; Dehaene, 1993; Nieder, 2016; Nieder & Dehaene, 2009; Piazza, 2011; Piazza et al., 2007). Indeed, if symbolic numerals are directly mapped onto their non-symbolic counterparts, we would expect compatible representations to positively interact in the congruent condition and incompatible representations to negatively interact in the incongruent condition. However, our data show that this was not the case. Therefore, our findings are in line with recent studies that question the existence of ANS and support the idea of separate processing mechanisms for symbolic and non-symbolic numerals (Marinova et al., 2021; Núñez, 2017; Sasanguie et al., 2015; Van Hoogmoed et al., 2021; Van Hoogmoed & Kroesbergen, 2018). Furthermore, our results suggest that non-symbolic numerals are fundamentally different from digits and comparable to other non-numerical magnitudes (Algom, 2021; Bar et al., 2019). This is supported by a previous review and meta-analysis, which showed that the effect size of the SNARC-like effect for non-numerical magnitudes is smaller than the effect size normally detected for symbolic numerals (Macnamara et al., 2018). Furthermore, this study also revealed a clear publication bias which suggests that non-significant results have not been published in studies investigating non-numerical magnitudes. Based on this evidence, it is not that surprising that symbolic numerals showed a clear SNARC effect in our study while non-symbolic numerals failed to do so.

A possible limitation of our study is that, from a perceptual point of view, symbolic and non-symbolic numerals were processed at different levels. Indeed, digits were processed at a local level while numerosity was processed at a global level (see Navon, 1977). This could be a potential confound in our design although there is no evidence that this phenomenon did affect the presence/absence of spatial–numerical associations in our study. Navon (1977) clearly showed that the processing of global features of a visual pattern preceded the one of local features and that only global information interferes with the processing of local targets. Thus, we should expect numerosity to interfere with the SNARC effect for digits, which was clearly not the case in our study. Furthermore, the absence of a SNARC effect for numerosity could not be ascribed to an interference due to digits, as global precedence should be unaffected by local information. Therefore, we are confident that our results have not been influenced by this phenomenon. Nevertheless, future studies should investigate the role of global precedence in the SNARC effect. Indeed, while numerosity can only be processed globally, digits could be combined to be processed both at a local and global level, thus allowing to systematically investigate this phenomenon.

Future studies should also consider using different non-symbolic configurations, numerosity range, and tasks. In the current study we tested non-symbolic numerals by employing a familiar structured configuration and a narrow range of stimuli, mostly within the subitizing range. There were three main reasons for this decision. Firstly, the numerosity employed (range 1–5) is consistent with that of dice patterns; secondly, there is evidence that the SNARC effect for non-symbolic numerals is stronger in the subitizing range (Mitchell et al., 2012); and finally, having the combination of a narrow range and a structured configuration fosters quick processing of numerosity and makes it comparable to that of digits. However, it would be interesting to know if our findings could be replicated with numerosity outside of the subitizing range and employing digits in random positions. The latter point would disrupt the meaningfulness of the global figure and would help to further investigate the effect of global precedence (Navon, 1977). Finally, as different task demands produced different results in some contexts, our findings should be replicated with different SNARC tasks, such as parity judgment.

To conclude, our results support recent evidence in favour of two independent processing systems for symbolic and non-symbolic numerals (Marinova et al., 2021; Sasanguie et al., 2015) and are in line with studies suggesting a fundamental distinction between these two numerical formats (Algom, 2021; Bar et al., 2019). Our study provides challenging evidence for the ANS theory, as well as the ATOM (Walsh, 2003) and the WM model (van Dijck & Fias, 2011).

### Supplementary Information

ESM 1 (DOCX 19 kb)

## Data Availability

All data and codes have been made publicly available at the Opens Science Framework and can be accessed online (https://osf.io/e7rj3/).

## Appendix

**Table 1:** Mean reaction times and standard errors for the symbolic task

Congruency	Response hand	Number magnitude	Mean (ms)	*SE*
Congruent	Left	Large	516.79	5.58
Congruent	Left	Small	492.43	4.37
Congruent	Right	Large	489.45	4.75
Congruent	Right	Small	509.73	5.67
Incongruent	Left	Large	542.55	6.54
Incongruent	Left	Small	506.16	4.50
Incongruent	Right	Large	509.51	5.20
Incongruent	Right	Small	523.75	5.42

**Table 2:** Mean reaction times and standard errors for the non-symbolic task

Congruency	Response hand	Numerosity magnitude	Mean (ms)	*SE*
Congruent	Left	Large	486.76	4.78
Congruent	Left	Small	493.40	4.81
Congruent	Right	Large	469.49	4.41
Congruent	Right	Small	493.40	5.31
Incongruent	Left	Large	503.06	4.75
Incongruent	Left	Small	510.83	5.42
Incongruent	Right	Large	487.89	5.08
Incongruent	Right	Small	507.72	5.36
