# Supplementary material for: Contrasting symbolic and non-symbolic numerical representations in a joint classification task
Source: Psychon Bull Rev. 2023 Jan 17;30(4):1422–30. doi: 10.3758/s13423-023-02246-w (PMC10482780; doi:10.3758/s13423-023-02246-w)
Supplement: Supplementary file 1 — (DOCX 19 kb) [file 13423_2023_2246_MOESM1_ESM.docx]

**Supplementary materials**

*Table 1: ANOVA table for the symbolic task*

| *Effect* | *Df* | *F ratio* | ${\eta_{p}}^{2}$ | *p* |
| --- | --- | --- | --- | --- |
| *Hand* | *1, 51* | *2.52* | *.047* | *.119* |
| ***Magnitude*** | ***1, 51*** | ***4.96*** | ***.089*** | ***.030*** |
| ***Congruency*** | ***1, 51*** | ***25.06*** | ***.330*** | ***<.001*** |
| ***Hand * Magnitude*** | ***1, 51*** | ***7.53*** | ***.129*** | ***.008*** |
| *Hand * Congruency* | *1, 51* | *0.00* | *<.001* | *.959* |
| *Magnitude * Congruency* | *1, 51* | *1.99* | *.037* | *.165* |
| *Hand * Magnitude * Congruency* | *1, 51* | *0.56* | *.011* | *.456* |

*Table 2: Post-hoc t-tests investigating significant hand* magnitude interaction in the symbolic task*

| *Contrast* | *Estimate* | *Df* | *T ratio* | *p* |
| --- | --- | --- | --- | --- |
| ***left large - right large*** | ***31.01*** | ***51*** | ***2.89*** | ***.027*** |
| ***left large - left small*** | ***32.04*** | ***51*** | ***3.22*** | ***.011*** |
| *left large - right small* | *15.28* | *51* | *2.17* | *.142* |
| *right large - left small* | *1.03* | *51* | *0.24* | *.994* |
| *right large - right small* | *-15.73* | *51* | *-1.76* | *.304* |
| *left small - right small* | *-16.76* | *51* | *-1.91* | *.236* |

*Table 3: ANOVA table for the non-symbolic task*

| *Effect* | *Df* | *F ratio* | ${\eta_{p}}^{2}$ | *p* |
| --- | --- | --- | --- | --- |
| ***Hand*** | ***1, 51*** | ***5.13*** | ***.091*** | ***.028*** |
| ***Magnitude*** | ***1, 51*** | ***13.32*** | ***.207*** | ***<.001*** |
| ***Congruency*** | ***1, 51*** | ***28.44*** | ***.358*** | ***<.001*** |
| *Hand * Magnitude* | *1, 51* | *0.58* | *.011* | *.449* |
| *Hand * Congruency* | *1, 51* | *0.12* | *.002* | *.726* |
| *Magnitude * Congruency* | *1, 51* | *0.39* | *.008* | *.537* |
| *Hand * Magnitude * Congruency* | *1, 51* | *0.38* | *.007* | *.542* |

*Note: Post-hoc t-tests were not conducted because no interaction was significant.*
